# Temporal patterns of multi-morbidity in 570157 ischemic heart disease patients: a nationwide cohort study

**DOI:** 10.1186/s12933-022-01527-3

**Published:** 2022-05-31

**Authors:** Amalie D. Haue, Jose J. Almagro Armenteros, Peter C. Holm, Robert Eriksson, Pope L. Moseley, Lars V. Køber, Henning Bundgaard, Søren Brunak

**Affiliations:** 1 Novo Nordisk Foundation Center for Protein Research, Faculty of Health and Medical Sciences, University of Copenhagen, Blegdamsvej 3B, 2200 Copenhagen, Denmark; 2 Department of Cardiology, The Heart Center, Rigshospitalet, Blegdamsvej 9, 2100 Copenhagen, Denmark; 3 Department of Infectious Diseases, Karolinska University Hospital, 171 76 Stockholm, Sweden; 4 Department of Clinical Medicine, Faculty of Health and Medical Sciences, University of Copenhagen, Blegdamsvej 3B, 2200 Copenhagen, Denmark; 5 Copenhagen University Hospital, Rigshospitalet, Blegdamsvej 9, 2100 Copenhagen, Denmark; 6 College of Health Solutions, Arizona State University, Arizona State University, 550 N 3rd St., Phoenix, AZ 85004 USA

**Keywords:** Ischemic heart disease, Multi-morbidity, Disease trajectories, Nationwide cohort study

## Abstract

**Background:**

Patients diagnosed with ischemic heart disease (IHD) are becoming increasingly multi-morbid, and studies designed to analyze the full spectrum are few.

**Methods:**

Disease trajectories, defined as time-ordered series of diagnoses, were used to study the temporality of multi-morbidity. The main data source was The Danish National Patient Register (NPR) comprising 7,179,538 individuals in the period 1994–2018. Patients with a diagnosis code for IHD were included. Relative risks were used to quantify the strength of the association between diagnostic co-occurrences comprised of two diagnoses that were overrepresented in the same patients. Multiple linear regression models were then fitted to test for temporal associations among the diagnostic co-occurrences, termed length two disease trajectories. Length two disease trajectories were then used as basis for constructing disease trajectories of three diagnoses.

**Results:**

In a cohort of 570,157 IHD disease patients, we identified 1447 length two disease trajectories and 4729 significant length three disease trajectories. These included 459 distinct diagnoses. Disease trajectories were dominated by chronic diseases and not by common, acute diseases such as pneumonia. The temporal association of atrial fibrillation (AF) and IHD differed in different IHD subpopulations. We found an association between osteoarthritis (OA) and heart failure (HF) among patients diagnosed with OA, IHD, and then HF only.

**Conclusions:**

The sequence of diagnoses is important in characterization of multi-morbidity in IHD patients as the disease trajectories. The study provides evidence that the timing of AF in IHD marks distinct IHD subpopulations; and secondly that the association between osteoarthritis and heart failure is dependent on IHD.

**Supplementary Information:**

The online version contains supplementary material available at 10.1186/s12933-022-01527-3.

## Background

Ischemic heart disease (IHD) is a common, chronic, multifactorial disease, and among the leading causes of death worldwide [1]. Up to 85% of IHD patients are diagnosed with other chronic diseases, which may impact the disease course and severity [2]. The cardiovascular risk reduction, including IHD, in patients with non-insulin-dependent diabetes treated with certain glucose-lowering drugs is evidence that multi-morbidity covers a phenotypic spectrum, where conventional diagnostics may fall short [3, 4]. While the literature within the single-disease paradigm is extensive, studies designed to analyze the full multi-morbidity spectrum are few [5–9]. However, such studies are becoming increasingly important, as improved survival among cardiovascular patients has stagnated; possibly due to the high and increasing incidences of other chronic diseases in these patients [10, 11].

Most common chronic diseases such as IHD and atrial fibrillation (AF), conditions related to the metabolic syndrome, and osteoarthritis (OA) are diagnosed within few years making it non-trivial to unmask true etiology [12–14]. For example, the risk of developing AF doubles for every decade of advanced age, while AF is also a major risk indicator after myocardial infarction [15, 16]. Studies designed to analyze the degree of association between IHD and OA present conflicting results [14, 17]. Moreover, results of the Framingham Heart Study have suggested that IHD, rather than hypertension and valvular disease, is the most common cause of heart failure (HF), which further substantiates the complexity of the order with which chronic diseases develop [18]. Yet, clinical management of IHD often comes down to absence or presence of risk factors and co-morbidities, leaving out information related to the temporal order of diagnoses and the disease history as a whole [12, 19, 20].

Here, we present a study set out to characterize the entire multi-morbidity landscape in IHD by means of temporal disease trajectories, defined as time-ordered series of diagnoses mapped at nationwide scale over a period of 24 years. Disease trajectories were originally developed as an approach for studying disease progression patterns comprehensively in the setting of nationwide register data and have recently been expanded to also analyze prescription data [21, 22]. We argue that disease trajectories describing patients diagnosed with IHD represent an important strategy to overcome the limitations that the single-disease paradigm are facing within the complex spectrum of multiple, chronic diseases. Thus, our study showcases information related to the temporal order of diagnoses that is currently omitted from clinical patient characterization.

## Methods

### Data foundation and study population

The main data source was the Danish National Patient Registry (NPR), where healthcare data from all encounters with Danish hospitals have been recorded since 1977. The data include contact type (i.e. in-patient, out-patient, and emergency room visits), date of contact start (e.g. admission), date of discharge, diagnosis codes, and diagnosis type (e.g. primary codes that best describe the contact reason and secondary codes that complement the description of the contact) [23]. To obtain demographic data on patients such as date of birth, sex, and status (dead or alive), data from NPR was linked to the Danish Civil Registration System (CRS) via Civil Personal Register numbers [24]. Since 1994, diagnoses in NPR have been reported using the International Statistical Classification of Diseases and Related Health Problems 10th Revision (ICD-10), which has a hierarchical structure comprising chapters, code blocks, level 3, and level 4 codes [25, 26]. The NPR dataset used in this study covers the period 1994–2018 and contains data from 7,179,538 individuals corresponding to more than 142 million contacts. There are 4565 distinct level 3 ICD-10 codes, which we here refer to as ICD-10 codes. Prior to analysis, level 4 codes were truncated to level 3 codes. Patients who were deceased by the end of the study were given the code Y99 and date of death was obtained from CRS [24].

**Fig. 1:**
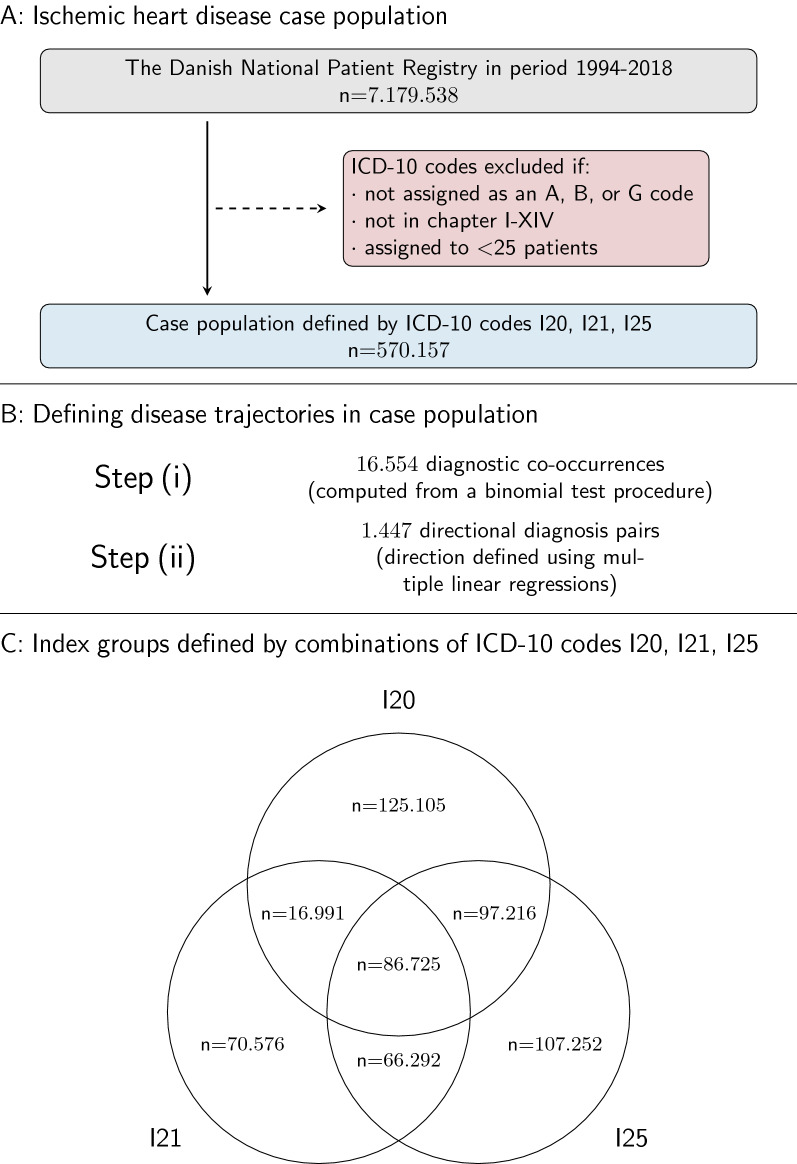
Study flowchart. **A** Identification of case population. Preprocessing of nationwide register data to construct disease trajectories by identification of directional disease pairs. Grey: Identification. Blue: Screening. Red: Exclusion. **B** Computation of disease trajectories (see text for details). **C** Definition of seven index groups. IHD: Ischemic heart disease. I20: Angina pectoris. I21: Acute myocardial infarction. I25: Chronic ischemic heart disease. ICD-10: International Statistical Classification of Disease and Health Related Problems 10th Revision

To define the case population, all patients in NPR who had been assigned an ICD-10 code for angina pectoris (ICD-10 code: I20), acute myocardial infarction (ICD-10 code: I21), or chronic IHD (ICD-10 code: I25) in the period 1994–2018 were first identified. All ICD-10 codes from chapters I-XIV assigned as a primary or secondary code (i.e., diagnosis types A, B, or G) to at least 25 patients were included. Next, patients who were assigned either of the diagnosis codes I20, I21, or I25 before the age of 18 years were excluded. Emigrants and tourists were also excluded, as their contacts with the Danish healthcare system are likely to be sporadic and thus, data for these patients are generally not available for the entire study period. Date of discharge in NPR was used to estimate age at diagnosis via linkage to CRS (Fig. 1).

### Experimental model and identification of diagnostic co-occurrences

To study the temporal order of multi-morbidities in the case population (i.e., patients diagnosed with IHD), directional diagnosis pairs were computed. Next, the directional diagnosis pairs were extended to trajectories comprised of three diagnoses [21]. The two main steps in computation of disease trajectories are (i) quantification of the overrepresentation of diagnostic co-occurrences between two diagnoses using relative risks (RRs) and (ii) identification of directional diagnostic co-occurrences where the temporal order of assignment is statistically significant (i.e., directional diagnosis pairs).

The first step (i) consists of a binomial test procedure that identifies pairs of diagnoses that co-occur in more patients than expected based on mean probability parameters specific for all diagnoses. For example, the procedure tests if HF is assigned to more patients with acute myocardial infarction compared to patients without a diagnosis code for acute myocardial infarction. For each diagnosis in NPR assigned to a minimum of 25 patients in the case population, the test procedure creates sets of exposed patients (e.g., patients assigned a diagnosis for acute myocardial infarction) and comparison patients (e.g., patients who were not assigned a diagnosis for acute myocardial infarction). For each pair of diagnoses being tested, 10,000 comparison groups are formed by sampling from un-exposed patients that are matched by sex, year of birth, and week of discharge to conservatively correct for e.g., seasonal variation in diagnosis codes and changes in coding practices. As our sample size consists of more than 19 million contacts, we can afford 10 000 comparison groups for each test (see Jensen et al. 2014 for details). By considering each discharge as a Bernoulli sample, the test procedure identifies diagnosis pairs that are significantly often assigned to the same patients compared to the mean probability parameter for the diagnosis considered exposed. Finally, RRs are calculated for all diagnosis pairs, e.g., acute myocardial infarction and HF. The RRs express the strength of the association between exposed patients (e.g. acute myocardial infarction) being diagnosed with some disease (e.g. HF) within five years, compared to unexposed patients. The level of significance was set to 0.001 to guard against false positives due to the binomial test procedure and corrected for multiple testing using the Bonferroni method. Scripts were run using R v. 3.4.0, Python v. 2.7, Python v. 3 and C +  + v. 11 [21, 27].

### Definition of directional diagnosis pairs and construction of disease trajectories

The second step (ii) establishes the directionality of diagnostic co-occurrences. In contrast to previous versions of the disease trajectory program, a series of multiple linear regression models (MLRs) was introduced. MLRs were introduced to identify diagnostic co-occurrences with a statistically significant difference between age at diagnosis, while adjusting for potential confounding factors (described below). In cases where the same diagnosis was assigned to a patient multiple times, only the earliest recorded diagnosis (with reference to discharge or end of contact) was included. In cases where a patient had more than one diagnosis assigned for the first time at the same contact, all codes were included in the regression analysis.

The dependent variable for the MLRs was age at diagnosis and the independent variables were the diagnosis pair from step (i), the type of diagnosis (primary—type A—or non-primary diagnosis—type B or G), discharge date, type of patient (in-patient or out-patient), and sex. These covariates were included to account for the possibility of differences in baseline characteristics at diagnosis due to factors not related to the natural course of disease development (i.e., sequence), e.g., changes in coding practice over the years. The P-value of the main effect for the diagnosis pair variable was used to determine the significance of difference in age between diagnosis D1 and diagnosis D2. The fitted age at D1 and D2 defined disease directionality, where D1 would be assigned at the youngest age. P-values were corrected using the Bonferroni method, setting the number of tests equal to the number of regressions. Significance level was set to 0.05. The MLRs were applied to all diagnostic co-occurrences identified in step (i) and fitted using statsmodels in Python 3.6.10 [28, 29].

Due to the number of covariates, it would be too demanding to obtain a fitted age for each subgroup, e.g., primary diagnosis, females, and outpatients for each discharge year. Therefore, the fitted age was calculated using only the coefficients for diagnosis pairs and type of diagnosis, as we observed that the covariate for the diagnosis type generally had the highest impact on the age difference and primary diagnoses appeared first (negative coefficient) for most of the diseases (Additional file 1: Figure S1). The fitted age was calculated for the two diagnoses when assigned as primary code and the diagnosis with the youngest fitted age was defined as the first diagnosis in the directional diagnosis (equivalent to length two trajectories) pair, which we represented using D1 → D2. The fit of models for the most relevant diagnosis pairs were evaluated where the distribution of the residuals was acceptable.

### Establishing disease trajectories of three diagnoses

To determine the directionality of disease trajectories containing three diagnoses, a similar set of MLRs was established based on patients who were assigned three diagnoses. Diagnosis pairs with a significant directionality where the second diagnosis of one pair was equal to the first diagnosis of another pair were pieced together into a length three trajectory (i.e., D1 → D2 and D2 → D3 into D1 → D2 → D3). The directionality of such a length three disease trajectory was determined by extracting all patients with the three diagnoses and calculating the fitted ages for the those diagnoses. The age at diagnosis was calculated using the set of three diagnoses (i.e., D1, D2, and D3) and type of diagnosis when assigned as primary diagnosis (Additional file 1: Figure S1). The diagnoses were ordered by estimated age, from youngest to oldest age, e.g., the length three trajectory D1 → D2 → D3. As fitted age at diagnosis was calculated separately for length two and three trajectories, the order of the same two diagnoses could be reversed in length two and length three trajectories, respectively. In addition, length three trajectories could establish new directional associations, as their assemblage did not require the first and the last diagnoses to be a directional diagnosis pair. That is, the trajectory D1 → D2 → D3 did not require a statistically significant association between diagnoses D1 and D3. In the final analysis, only trajectories computed based on sets of more than 50 patients were included; and patients with diagnoses D1 and D2 or D1, D2 and D3 were then said to follow the resulting trajectory, of length two and three, respectively.

### Characterization of different IHD populations based on disease trajectories

Finally, to compare different IHD subgroups based on disease trajectories, the cohort was split into seven subgroups defined by assigned IHD codes (Fig. 1). Three groups were defined by having only I20, I21, or I25. Another three groups were defined by having two of the three codes, e.g. I20 and I21. A final group was defined by patients that were assigned I20, I21 and I25. These groups comprised a total of seven distinct index groups. Disease trajectories were computed for each of these groups separately, following the previously described step (ii).

## Results

### Characterization of the IHD case population using index subgroups

A total of 570,157 patients (57.5% males) diagnosed with IHD by ICD-10 codes I20 (angina pectoris), I21 (acute myocardial infarction) or I25 (chronic ischemic heart disease) during 1994–2018 were included in the study. Mean age at first IHD diagnosis was 65.9 years for males and 70.9 years for females. At the end of the study period, 54.3% of the population were dead (52.1% for males and 57.1% for females). As expected, essential hypertension (I10), non-insulin-dependent diabetes (E11), insulin-dependent diabetes (E10), AF (I48), HF (I50) and dyslipidemia (E78), were among the co-morbidities with the highest prevalence in the IHD cohort. Except for pneumonia (J18) and cystitis (N30), diagnoses that were prevalent in the cohort were generally chronic conditions or manifestations of chronic diseases (Table 1).

**Table 1:** Population characteristics and the 15 co-morbidities assigned to most patients

	Patients (n)	Mean age at IHD in years (SD)	Patients dead at end of study		Mean number of directional diagnosis pairs
			n	%	
Total	570,157	68.0 (13.7)	309,326	54.3%	25.1
Males	327,876	65.9 (13.2)	170,942	52.1%	23.8
Females	242,281	70.9 (13.9)	138,384	57.1%	25.4
*Index groups (ICD-10 code)*					
Angina pectoris (I20)	125,105	62.4 (14.2)	38,861	31.1%	14.4
Acute myocardial infarction (I21)	70,576	72.7 (14.2)	51,502	73.0%	12.4
Chronic ischemic heart disease (I25)	107,252	74.9 (12.3)	78,762	73.4%	24.2
Angina pectoris (I20) Acute myocardial infarction (I21)	16,991	67.3 (13.9)	9,665	56.9%	20.3
Angina pectoris (I20) Acute myocardial infarction (I25)	97,216	66.4 (11.4)	46,062	47.4%	32.3
Acute myocardial infarction (I21) Chronic ischemic heart disease (I25)	66,292	69.1 (12.9)	36,635	55.3%	24.3
Angina pectoris (I20) Acute myocardial infarction (I21) Chronic ischemic heart disease (I25)	86,725	65.0 (12.1)	47,456	54.7%	41.4
*Diagnoses (ICD-10 code)*					
Hypertension (I10)	251,032	67.7 (12.4)	118,118	20.7%	36.4
Heart failure (I50)	167,863	72.2 (12.0)	125,771	22.1%	39.4
Dyslipidemia (E78)	155,707	63.3 (11.5)	54,683	9.6%	35.5
Atrial fibrillation (I48)	147,896	73.5 (11.6)	99,682	17.5%	39.0
Pneumonia (J18)	142,183	71.9 (12.4)	105,826	18.6%	42.0
Senile cataract (H25)	126,008	74.0 (10.9)	75,022	13.2%	39.2
Non-insulin-dependent diabetes (E11)	101,822	67.2 (12.3)	59,604	10.5	45.1
Other hearing loss (H91)	101,415	74.6 (11.7)	64,340	11.3%	35.1
Other chronic obstructive pulmonary disease (J44)	95,260	70.2 (11.0)	67,502	11.8%	42.5
Cystitis (N30)	80,088	73.3 (12.5)	55,829	9.8%	45.4
Dorsalgia (M54)	65,828	63.7 (14.1)	25,718	4.5%	38.2
Stroke (I64)	61,763	72.4 (11.8)	47,526	8.3%	42.5
Anemia (D64)	61,373	73.3 (11.8)	46,418	8.2%	50.7
Other disorders of the urinary system (N39)	59,581	71.8 (12.4)	37,360	6.6%	49.0
Atherosclerosis (I70)	59,087	70.5 (11.4)	44,895	7.9%	48.1

Based on combinations of the ICD-10 codes I20, I21 and I25 we defined seven index subgroups of the case population (Fig. 1). The size of the seven index groups ranged from 16,991 to 125,105 patients. The group defined by angina pectoris (I20) only was the largest of the index groups and patients in this group were youngest when diagnosed with IHD (mean age at diagnosis: 62.4 years) (Table 1). Using the entire set of diagnosis codes assigned to patients in the case population, a total of 16,554 diagnostic co-occurrences were identified. Among all the diagnostic co-occurrences, there were 1447 pairs with a statistically significant difference between mean age at the two diagnoses (Fig. 1).

### Characterization of the multi-morbidity landscape by means of disease trajectories

**Fig. 2:**
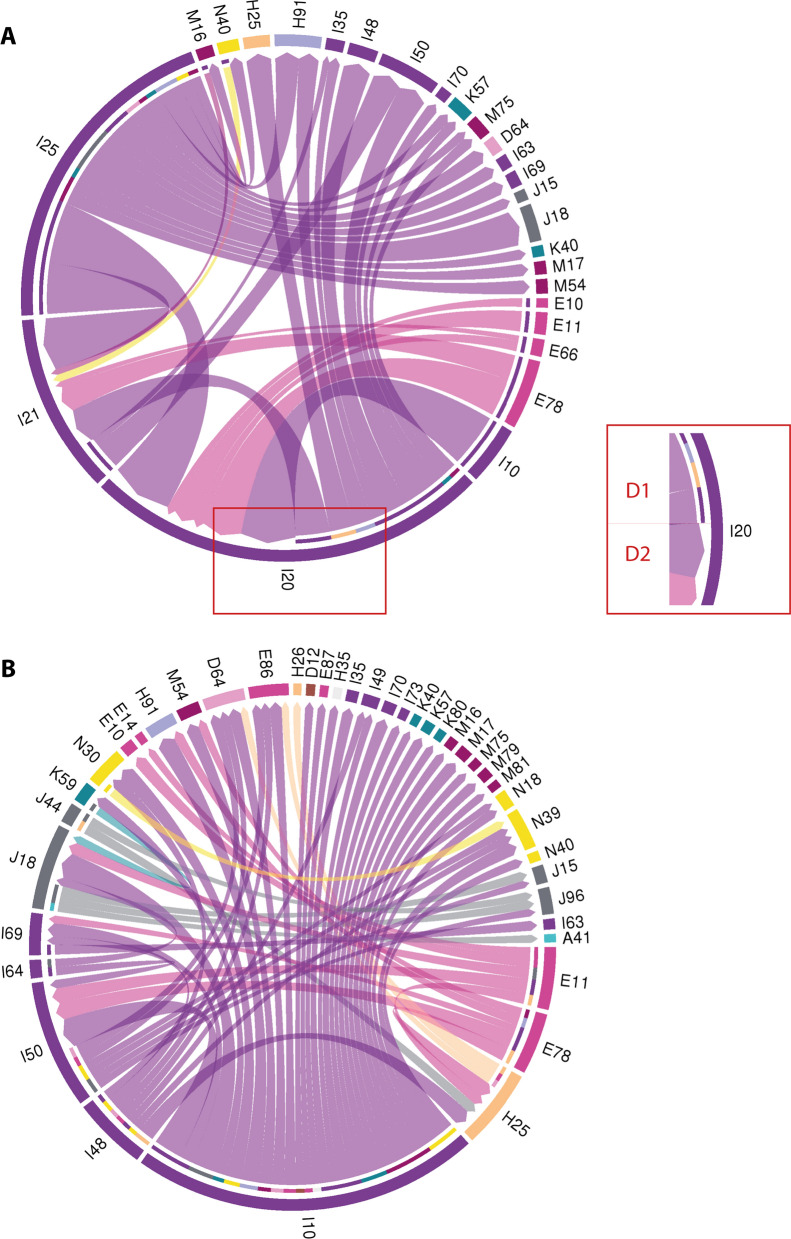
Graphical summary of length two disease trajectories. Chord diagrams displaying the directional relations for selected directional diagnosis pairs. ICD-10 codes that comprise directional pairs are marked in the periphery and ribbons indicate directional diagnosis pairs. Width of ribbons corresponds to the number of patients that follow a directional diagnosis pair. **A** The 35 directional diagnosis pairs followed by most patients that contain at least one ICD-10 code for IHD. Number of patients represented: 469,342. Diagnosis D1 refers to the diagnosis that appears first (youngest age) and diagnosis D2 refers to the diagnosis D2 (oldest age) in the directional diagnosis pair as indicated in the insert on right. **B** All directional diagnosis pairs that did not contain an ICD-10 code for IHD and followed by more than 20,000 patients. Number of patients represented: 355,921. Number of directional diagnosis pairs: 63. Diagnoses D1 and D2 are indicated as they are in A. **A and B**: Number of distinct patients in A and B: 331,207. Only directional diagnoses pairs without Y99 (death) and more than three months between estimated age at D1 and D2 are depicted. Color key according to ICD-10 chapter and available in Additional file 1: Figure S3. IHD: Ischemic heart disease. ICD-10: International Statistical Classification of Disease and Health Related Problems 10th Revision. For a full list of ICD-10 codes and descriptions see Additional file 1: Table S1

The 1 447 directional diagnosis pairs (i.e., length two trajectories) contained a total of 459 distinct ICD-10 codes. The temporal associations between IHD and other chronic conditions were highly diverse and dominated by other diseases of the cardiovascular system as well as metabolic diseases. In the most common trajectories, chronic IHD (I25) primarily appeared as D1, whereas angina pectoris (I20) appeared as D1 as well as D2 (Fig. 2A). Interestingly, insulin-dependent diabetes (E11) and non-insulin-dependent diabetes displayed a similar pattern in relation to IHD (Fig. 2A), whereas they differed in their temporal association to other diagnoses than IHD (Fig. 2B). Piecing together length two trajectories and presenting them as a single connected graph illustrated nicely the multi-morbidity landscape in IHD (Additional file 1: Figure S2). In the 1447 length two disease trajectories, hypertension (I10) was the most common diagnosis, occurring in 129 of the trajectories; and in 126 of these, hypertension appeared as D1 consistent with the fact that hypertension (I10) is primarily a risk factor, rather than a disease complication. The same trend was observed for both non-insulin-dependent diabetes (E11), insulin-dependent diabetes (E11), and AF (I48) that appeared as D1 in more than 75% of the cases. In contrast, the distribution of the first and second diagnosis was more even for angina pectoris (I20), HF (I50), acute myocardial infarction (I21), and cystitis (N30). Diagnoses such as osteoporosis without pathological fracture (M81) and diverticular disease of the intestine (K57) were examples of diagnoses that primarily appeared as D2, indicating that they are rarely the first manifestation of multi-morbidity. For some directional diagnosis pairs, we observed that the total number of patients following them was roughly the same as that of either of the two diagnoses, meaning that all patients with these diagnoses had other diagnoses that associated in a temporal manner (e.g., hypertension and osteoporosis) (Table 2).

**Table 2:** Diagnoses that appear in at least 25 directional diagnosis pairs (D1 → D2)

ICD-10	Description	Number of trajectories per diagnosis			Number of distinct patients per trajectory with diagnosis		
		Total	D1	D2	Total	D1	D2
I10	Hypertension	129	126	6	249,797	249,652	44,326
I25	Chronic ischemic heart disease	104	93	11	355,100	354,669	61,979
E11	Non-insulin-dependent diabetes	90	89	1	101,507	101,493	2,525
I20	Angina pectoris	71	46	25	316,688	293,978	280,966
I48	Atrial fibrillation	56	42	10	146,221	139,275	102,242
E10	Insulin-dependent diabetes	45	44	1	43,243	43,140	34,656
I50	Heart failure	52	23	29	167,838	149,446	159,595
E78	Dyslipidemia	52	47	5	155,389	155,324	28,788
E66	Obesity	42	42	0	44,324	44,324	0
M62	Other disorders of muscle	41	41	0	38,598	38,598	0
I21	Acute myocardial infarction	40	14	26	235,633	187,056	200,793
E86	Volume depletion	39	1	38	58,469	47,417	56,836
H25	Senile cataract	37	13	24	124,799	104,124	117,541
I46	Cardiac arrest	30	1	31	20,263	16,583	20,263
N92	Excessive, frequent, and irregular menstruation	29	28	1	14,503	14,503	479
M81	Osteoporosis without pathological fracture	29	7	22	37,727	28,832	37,727
K57	Diverticular disease of intestine	28	3	25	46,705	27,008	46,189
J96	Respiratory failure, not elsewhere classified	28	1	27	40,819	32,119	40,457
J44	Other chronic obstructive pulmonary disease	28	25	3	89,885	88,625	16,051
D64	Other anemias	27	1	26	61,109	46,546	60,239
N30	Cystitis	26	13	13	78,603	70,081	70,428
J42	Unspecified chronic bronchitis	25	25	0	22,546	22,546	0
I69	Sequelae of cerebrovascular disease	25	15	10	56,998	54,771	54,410

A quantitative summary of the directional diagnosis pairs revealed additional characteristics regarding multi-morbidity in IHD that were not captured by the crude counts of diagnoses. For example, insulin-dependent diabetes (E10) and obesity (E66) were among the most frequently occurring diagnoses in the directional diagnosis pairs, although they were not among the diagnoses assigned to most patients in the population. Similarly, osteoporosis without pathological fracture (M81) and diverticular disease of the intestine (K57) were among the diagnoses that associated with most diagnoses in a directional manner, albeit not among the most prevalent diagnoses in the population. Conversely, conditions that are not chronic, such as pneumonia (J18) and cystitis (N30), were among the diagnoses assigned to most patients, yet they were not among the most frequently occurring diagnoses in the directional diagnosis pairs (Tables 1, 2).

### Information obtained from a comparison of length two and length three trajectories

**Fig. 3:**
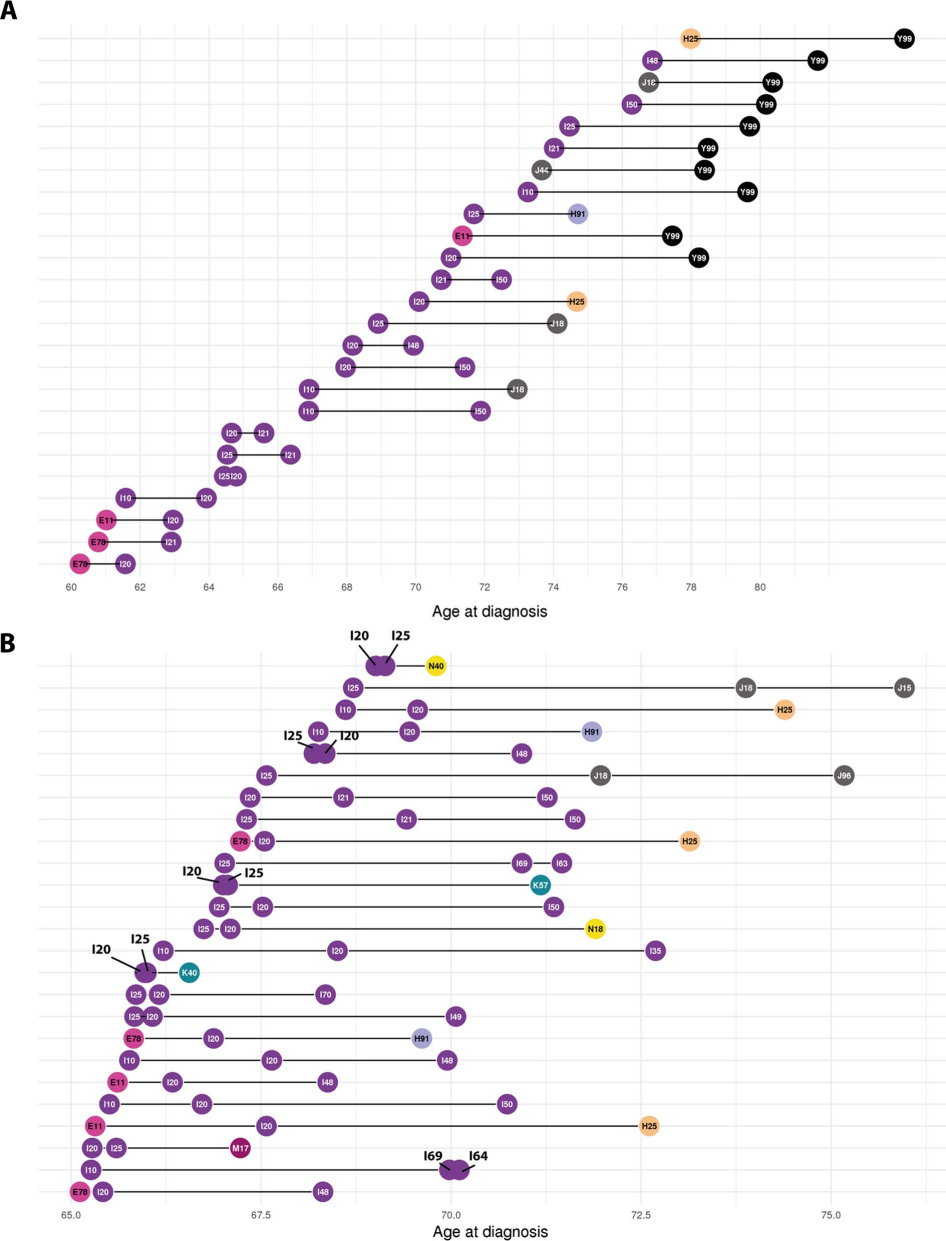
Overview of selected length two and three trajectories ordered by age at diagnoses. **A** Plot illustrating the mean age at diagnoses D1 and D2 for the 25 directional diagnosis pairs that most patients followed arranged by mean age at D1 (in descending order going down on Y-axis). Each horizontal line segment corresponds to a length two trajectory. **B** Plot illustrating the mean age at diagnoses D1, D2, and D3 for the 25 length three trajectories followed by most patients where fitted age at D1 is between 65 and 70 years and who weren't disease by the end of the study (absence of Y99). X-axis: Age in years (continuous). Y-axis: Length two (**A**) or three (**B**) trajectories. ICD-10: International Statistical Classification of Disease and Health Related Problems 10^th^ Revision. Color key according to ICD-10 chapter and available in Additional file 1: Figure S3. Circles indicate diagnoses. Diagnosis D1 is a diagnosis that appears at the earliest age and will be represented furthest to the left. For a full list of ICD-10 code definitions see Additional file 1: Table S1

Next, the 1447 length two disease trajectories were combined into 4729 length three trajectories, i.e., disease trajectories comprised of three diagnoses (for details, see “Methods” section). Selected length two and three disease trajectories with shared diagnoses were then compared. Generally, the fitted ages for IHD in trajectories containing IHD risk factors, e.g., dyslipidemia (E78) and hypertension (I10) were younger than trajectories containing a diagnosis code for IHD and no risk factors. In contrast, among dead patients (Y99), the fitted age at IHD was higher primarily reflecting that this instance generally captured older patients. However, in trajectories that contained death (Y99) and a code for common IHD risk factors e.g., E78 or I10, age at death was generally lower (Fig. 3).

For some diagnoses the fitted age at diagnosis varied considerably between trajectories. For example, the fitted age at angina pectoris (I20) was below 60.8 years for patients diagnosed with dyslipidemia and angina pectoris (i.e., E78 → I20, n = 114 071). In contrast, the fitted age at the diagnosis of angina pectoris (I20) was 68.0 years for patients diagnosed with angina pectoris and HF (i.e., I20 → I50, n = 84 952). When combined into a length three trajectory, i.e., E78 → I20 → I50 it was primarily the age at diagnosis of HF (I50) that changed. The fitted age at diagnosis of HF (I50) among patients diagnosed with angina was 71.4 years whereas it was 68.3 years for patients diagnosed with dyslipidemia (E78), angina pectoris (I20) and HF (Table 3).

**Table 3:** Summary of selected disease trajectories

Length two trajectories								
ICD-10 D1	ICD-10 D2		Age in years, D1 (95% CI)	Age in years, D2 (95% CI)	Adj. P	Counts	RR, D1, D2	
I10	I20		62.8 (62.7;63.0)	64.9 (64.9;65.0)	< 0.001	162,374	1.93	
I10	Y99		74.3 (74.0;74.1)	80.2 (80.2;80.3)	0	118,440	–	
E78	I20		61.3 (61.1;61.6)	62.5 (62.4;62.6)	< 0.001	114,071	4.36	
I20	I50		68.9 (68.9;69.0)	72.2 (72.2;72.5)	< 0.001	84,952	1.94	
I20	I48		69.0 (69.0;69.1)	70.8 (70.7;70.9)	< 0.001	80,558	1.34	
I21	I50		71.1 (71.3;71.9)	73.4 (73.3;73,6)	< 0.001	84,990	2,30	
M16	I21		71.5 (71.4;71.7)	72.3 (72.1;72.5)	< 0.001	17,316	2.62	

Length three trajectories								
ICD-10 D1	ICD-10, D2	ICD-10, D3	Age, D1	Age, D2	Age, D3	Counts	RR, D1, D2	RR, D2, D3
E78	I20	I50	63.7	63.9	68.3	32,309	4.36	1.94
E78	I20	I48	65.1	65.4	68.3	30,248	4.36	1.34
E78	I21	M16	66.1	67.7	68.6	6437	3.69	2.62
I10	I20	I25	62.9	64.6	64.7	105,652	1.93	4.28
M16	I21	I50	73.0	74.2	76.4	6760	2.62	2.30

### Disease trajectories identify temporal associations that depend on more than two diagnoses

We observed that length three trajectories captured temporal trends in the cohort that length two trajectories did not identify. For example, albeit the length three trajectory I10 → I20 → I25 was followed by 105,652 patients, the diagnoses I10 and I25 did not form a length two trajectory. The number of patients following the trajectory I10 → I20 → I25 corresponded to 66.1% of the patients following the trajectory I10 → I20 (n = 162 374, P < 0.001). This indicates that angina pectoris (I20) is essential for the temporal association between hypertension (I10) and chronic IHD (I25). A similar trend was observed when comparing dyslipidemia (E78) and AF (I48) (no diagnostic co-occurrence) with patients following the trajectory E78 → I20 → I48 (n = 32 248), corresponding to 28.3% of patients who followed the trajectory E78 → I20 (n = 114 071) (Table 3).

Among the length three trajectories we found the trajectory M16 → I21 → I50, indicating that acute myocardial infarction (I21) is essential for the association between OA of the hip and HF as no length two disease trajectory comprised of the diagnoses OA of the hip (M16) and HF (I50) was observed (Table 3). Further, in the population of patients who had been assigned the diagnosis code for OA of the hip (M16) and acute myocardial infarction (I21), the fitted age for OA of the hip was lower than that of acute myocardial infarction (fitted ages 70.9 years and 71.4 years, for M16 and I21, P < 0.001). However, among patients with the diagnosis code for dyslipidemia (E78), OA of the hip (M16) and acute myocardial infarction (I21) the order was reversed, i.e., the fitted age of I21 was younger than that of M16 as fitted ages were 68.6 and 67.7 for M16 and I21, respectively (Table 3). This indicates the dual nature of OA that might be a component of the metabolic syndrome, a marker of lack of mobility/reduced exercise, or simply age-related degeneration.

### Disease trajectories with different patterns in IHD subpopulations

Finally, we characterized selected disease trajectories in different index groups. Ages at HF (I50) and acute respiratory failure (J96) were similar in the index groups defined by patients diagnosed with angina pectoris (I20) and chronic IHD (I25); and patients diagnosed with angina pectoris (I20), acute myocardial infarction (I21), and chronic IHD (I25). Among patients indexed with I20 and I25, estimated age at HF (I50) was 70.5 years and 75.0 years for acute respiratory failure (n = 3 882, P < 0.0001). For patients indexed with I20, I21, and I25 it was 70.0 years and 74.8 years for HF and acute respiratory failure, respectively (n = 5 161, P < 0.001) (Table 4). These observations suggest that there is a clearer temporal association between heart failure (I50) and acute respiratory failure (J96) than between acute myocardial infarction (I21) and atrial fibrillation (I48).

**Table 4:** Summary of selected disease trajectories for different index populations

Index codes	Counts Total	ICD-10 D1	ICD-10, D2	Age, D1	Age, D2	Adj. P	Counts Trajectory	RR, D1, D2
I21	70,576	I48	I21	73.3	76.2	< 0.001	11,868	1.09
I20, I21, I25	86,725	I21	I48	69.4	71.2	< 0.001	26,273	1.09
I20, I25	97,216	I50	J96	70.5	75.0	< 0.001	3882	1.68
I20, I21, I25	86,725	I50	J96	70.0	74.8	< 0.001	5161	1.68

As noted above, AF (I48) most often appeared as the first diagnosis in length two trajectories, indicating that AF primarily occurred as an early manifestation of multi-morbidity in this population. However, in the length two disease trajectories with a diagnosis code for IHD and AF, the fitted age of IHD was lower than that of AF, e.g. I20 → I48 (n = 80 558, I20, age: 69.0, I48, age: 70.8, P < 0.001). Patients following this trajectory were among the patients that were oldest when diagnosed with angina pectoris (I20), indicating that AF is not associated with younger age at onset for angina pectoris (Table 3).

When the cohort was analyzed in its entirety, the diagnoses acute myocardial infarction (I21) and AF (I48) did not comprise a directional diagnosis pair meaning that there was no significant age difference between age at acute myocardial infarction and age at AF. However, there was a significant age difference between age at diagnosis in two index groups. For patients with a diagnosis code for acute myocardial infarction (I21) and neither code for angina pectoris (I20) nor chronic IHD (I25), mean age at diagnoses for AF was 73.3 years and 76.3 years for acute myocardial infarction, i.e. I48 → I21 (n = 11 871, P < 0.001). In contrast, the order was reversed, i.e., patients were younger when diagnosed with acute myocardial infarction when calculated for the population who was indexed with diagnosis codes I20, I21 and I25 (n = 26 273, P < 0.001) (Table 4).

## Discussion

We presented a strategy for analyzing the temporal order of IHD co-morbidities based on trends in nationwide register data from more than 500 000 IHD patients observed over a period of 24 years. By first establishing diagnostic co-occurrences and then piecing together disease trajectories, we present a comprehensive characterization of multi-morbidity in IHD centered on temporal associations. The disease trajectories captured temporal associations and interactions withinn multi-morbidity that are usually omitted in a purely hypothesis-driven studies. Generally, chronic conditions were more prevalent in disease trajectories as opposed to raw counts of co-morbidities, where both cystitis and pneumonia were common (Tables 1, 2). Previous studies have primarily analyzed IHD in relation to selected chronic diseases, such as AF, diabetes, or HF [16, 17]. In contrast, this study sheds light on the fact that the sequence of diagnoses for individual patients differs. In a clinical context, the sequence of diagnoses in multi-morbid patients is largely omitted from patient characterization. Here, we demonstrated at a nationwide scale that the sequence of diagnoses varies in different IHD subpopulations using the association between acute myocardial infarction and AF as an example. This finding is consistent with the dual nature of AF that may be an age phenomenon as well as a disease complication, including common conditions such as pulmonary embolism where AF is a frequent sequela. Moreover, we used the length three disease trajectories to identify associations of more than two diagnoses that would otherwise have been missed. For example, we found that the temporal association of dyslipidemia and AF required a diagnosis of angina pectoris prior to a diagnosis of AF. Such analysis calls for future focused studies assessing if it is true that in isolation, dyslipidemia is not a risk factor for AF, which again may call for differential antiarrhythmic therapies depending on the sequence of diagnoses leading to AF. As temporal interrelatedness beyond several risk factors for the same disease is largely omitted in patient characterization, temporal analyses of multi-morbidity may partly explain currently conflicting literature in this domain.

Finally, the disease trajectories served as a tool to identify cases where OA was more likely to be a component of metabolic syndrome as opposed to an age-related degeneration in a single organ system (Table 2). Similarly, the disease trajectories facilitated identification of OA as one of the co-morbidities that appeared in most directional diagnosis pairs, although it was not among the most common diagnoses in the population. Thus, such a diagnosis is likely to be underestimated within single-disease, cross-sectional studies. , the potential to link more than two diagnoses casts light on the immense heterogeneity within IHD. For example, we found that in this setup acute myocardial infarction is essential to establish an association between OA and HF. Potential clinical implications of these findings are better patient treatment facilitated by a refined understanding of the etiology within IHD multi-morbidity.

The study has several strengths and limitations. Generally, the disease trajectory approach offers a novel strategy to identify temporal trends and factor interactions in the complex multi-morbidity landscape of IHD. We argue that this strategy can complement traditional studies, where multi-morbidity is assessed in a binary fashion, depending on their presence or absence [13]. An inherent limitation with NPR (despite its long observation period) is that the disease history of the individual patient is not complete, meaning that all data is conditioned on the fact that the patient went to the hospital, but not necessarily hospitalized. Moreover, the diseases did not necessarily appear in the order they were registered. Similarly, there will of course be cases where the true age at first diagnosis was earlier than 1994 and hence will not be captured in the analysis. We assume that for patients with many contacts before 1994 this will apply to most diagnoses that are then likely to be registered at the same contact in the observation period. For patients with only few contacts before the start of the study that are close in time to 1994 this will only have limited impact on the results. Further, due to differences in year of birth for study participants combined with the broad inclusion criteria, differences in disease directionality may be confounded by factors not related to etiological differences. Ultimately, future studies will also include data from the Danish ICD-8 period, i.e., before 1994, and healthcare data from the primary care sector. In its current form, the method can only define a direction in a predefined population meaning that the direction is determined using the entire distribution for the population instead of the recorded sequence for the individual patient.

## Conclusions

The sequence of diagnoses is important in characterization of multi-morbidity in IHD patients as the disease trajectories. The study provides evidence that the timing of AF in IHD marks distinct IHD subpopulations; and secondly that the association between OA and HF is dependent on IHD. Further studies are needed to determine the actual order of diagnoses in the individual patient and thereby disentangle the true disease mechanism in cases where one condition may both appear as a risk factor and a complication. Ultimately prognostic individual patient models will be needed to develop more personalized treatment in the IHD domain. We argue that the value of studying nationwide health register data comprehensively outweighs the limitations and calls for future collaborations between basic and physician scientists.

## Supplementary Information

**Additional file 1: Fig. S1**. Distribution of coefficients for covariates in the multiple linear regressions. Regression coefficients for the covariates type of patient (in- or out-patient), type of diagnosis (primary or non-primary diagnosis code), and sex (male or female). One observation per evaluated diagnosis and all diagnoses were evaluated in each plot.** Fig. S2**. Disease trajectory network pieced together from length two trajectories. Circles represent diagnosis codes (ICD-10 codes), and arrows represent a length two trajectory. IHD risk factors such as type 2-diabetes (E11) and hypertension (I10) appear in the left side of the graph. Angina pectoris (I20), acute myocardial infarction (I21), and chronic IHD (I25) have many incoming as well as outgoing edges consistent with the wide phenotypic spectrum they represent. ICD-10: International Statistical Classification of Diseases and Related Health Problems 10th Revision. IHD: Ischemic heart disease. For a full list of ICD-10 code definitions see Table S1.** Fig. S3**. Color key according to ICD-10 chapter. ICD-10: International Statistical Classification of Diseases and Related Health Problems 10th Revision chapter I through XIV.** Table S1**. ICD-10 codes and descriptions.

## Data Availability

Permission to access and analyse the underlying person-sensitive data can be obtained following approval from the Danish Data Protection Agency and the Danish Health Authority.
