# Supplementary material for: Temporal patterns of multi-morbidity in 570157 ischemic heart disease patients: a nationwide cohort study
Source: Cardiovasc Diabetol. 2022 May 31;21:87. doi: 10.1186/s12933-022-01527-3 (PMC9158400; doi:10.1186/s12933-022-01527-3)

**Supplemental Material**

Short title: Disease trajectories in ischemic heart disease

Haue AD, Almagro Armenteros JAA, et al.

**Supplementary figure 1: Distribution of coefficients for covariates in the multiple linear regressions.** Regression coefficients for the covariates type of patient (in- or out-patient), type of diagnosis (primary or non-primary diagnosis code), and sex (male or female). One observation per evaluated diagnosis and all diagnoses were evaluated in each plot.

**Supplementary figure 2: Disease trajectory network pieced together from length two trajectories.** Circles represent diagnosis codes (ICD-10 codes), and arrows represent a length two trajectory. IHD risk factors such as type 2-diabetes (E11) and hypertension (I10) appear in the left side of the graph. Angina pectoris (I20), acute myocardial infarction (I21), and chronic IHD (I25) have many incoming as well as outgoing edges consistent with the wide phenotypic spectrum they represent. ICD-10: International Statistical Classification of Diseases and Related Health Problems 10^th^ Revision. IHD: Ischemic heart disease. For a full list of ICD-10 code definitions see supplementary table 1.

**Supplementary figure 3: Color key according to ICD-10 chapter.** ICD-10: International Statistical Classification of Diseases and Related Health Problems 10^th^ Revision chapter I through XIV.

**Supplementary table 1: ICD-10 codes and descriptions**

| **ICD-10 code** | **Description** |
| --- | --- |
| A41 | Other sepsis |
| D64 | Other anaemias |
| E10 | Type 1 diabetes mellitus |
| E11 | Type 2 diabetes mellitus |
| E14 | Unspecified diabetes mellitus |
| E66 | Obesity |
| E78 | Disorders of lipoprotein metabolism and other lipidaemias |
| E86 | Volume depletion |
| E87 | Other disorders of fluid, electrolyte and acid-base balance |
| G45 | Transient cerebral ischaemic attacks and related syndromes |
| H25 | Senile cataract |
| H26 | Other cataract |
| H91 | Other hearing loss |
| I10 | Essential (primary) hypertension |
| I20 | Angina pectoris |
| I21 | Acute myocardial infarction |
| I25 | Chronic ischemic heart disease |
| I35 | Nonrheumatic aortic valve disease |
| I48 | Atrial fibrillation and flutter |
| I49 | Other cardiac arrythmias |
| I50 | Heart failure |
| I63 | Cerebral infarction |
| I69 | Sequelae of cerebrovascular disease |
| I70 | Atherosclerosis |
| I73 | Other peripheral vascular diseases |
| J15 | Bacterial pneumonia, not elsewhere classified |
| J18 | Pneumonia, organism unspecified |
| J44 | Other chronic obstructive pulmonary disease |
| J96 | Respiratory failure, not elsewhere classified |
| K40 | Inguinal hernia |
| K57 | Diverticular disease of intestine |
| K59 | Other functional intestinal disorders |
| K80 | Cholelithiasis |
| M16 | Arthrosis of hip |
| M17 | Arthrosis of knee |
| M54 | Dorsalgia |
| M75 | Shoulder lesions |
| M79 | Other soft tissue disorders, not elsewhere classified |
| M81 | Osteoporosis without pathological fracture |
| N18 | Chronic kidney disease |
| N30 | Cystitis |
| N39 | Other disorders of the urinary system |
| N40 | Hyperplasia of prostate |


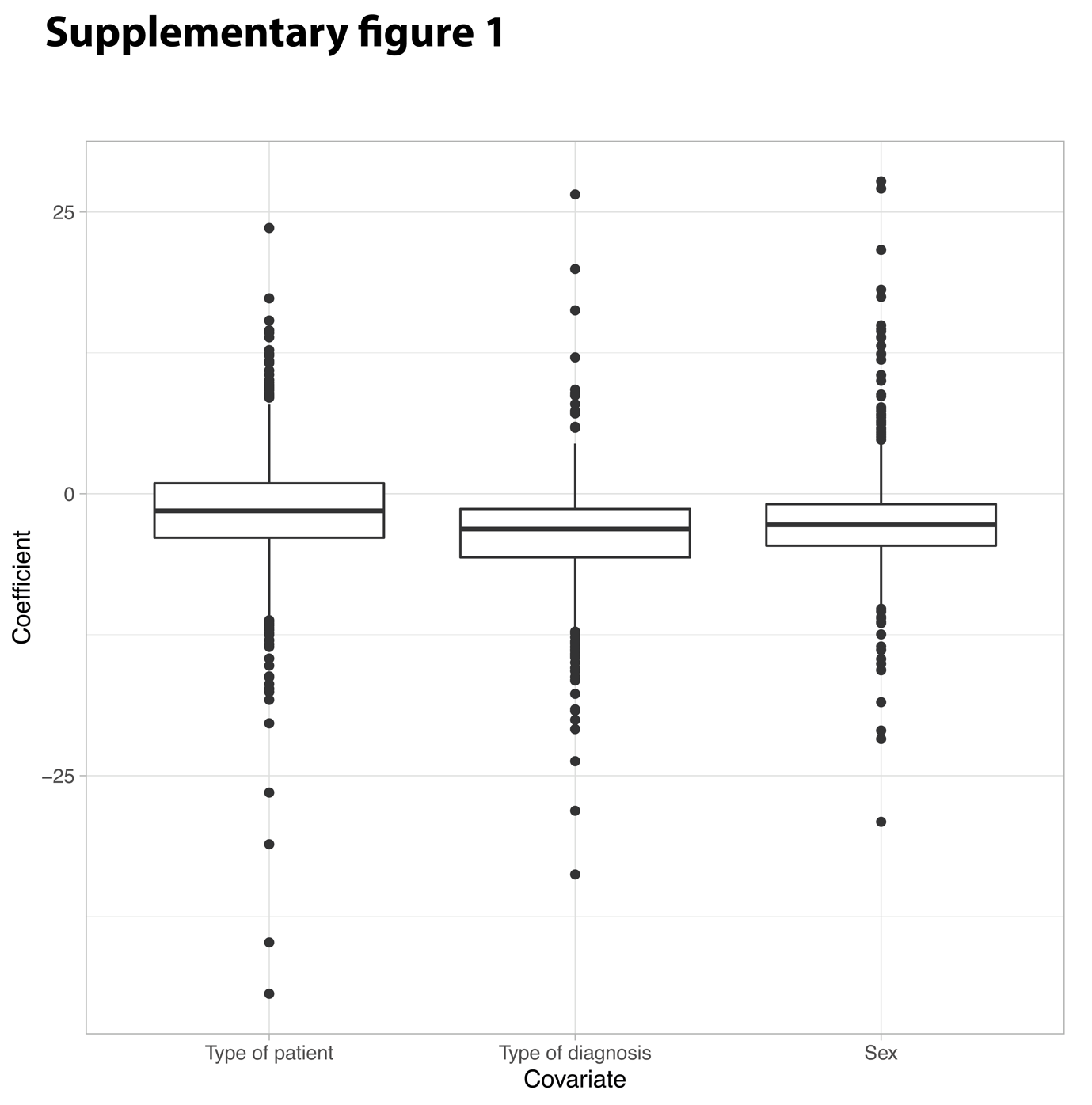


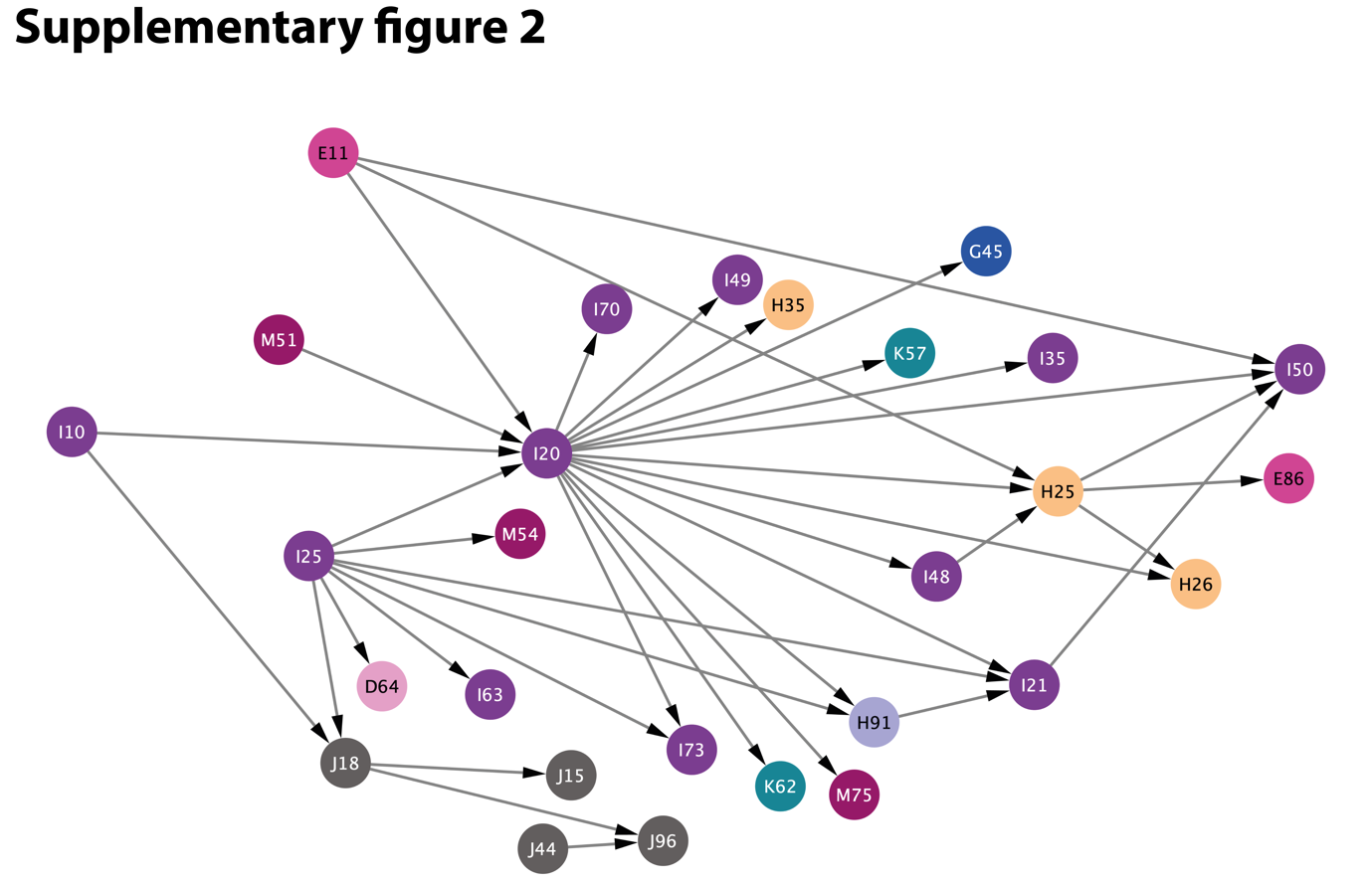


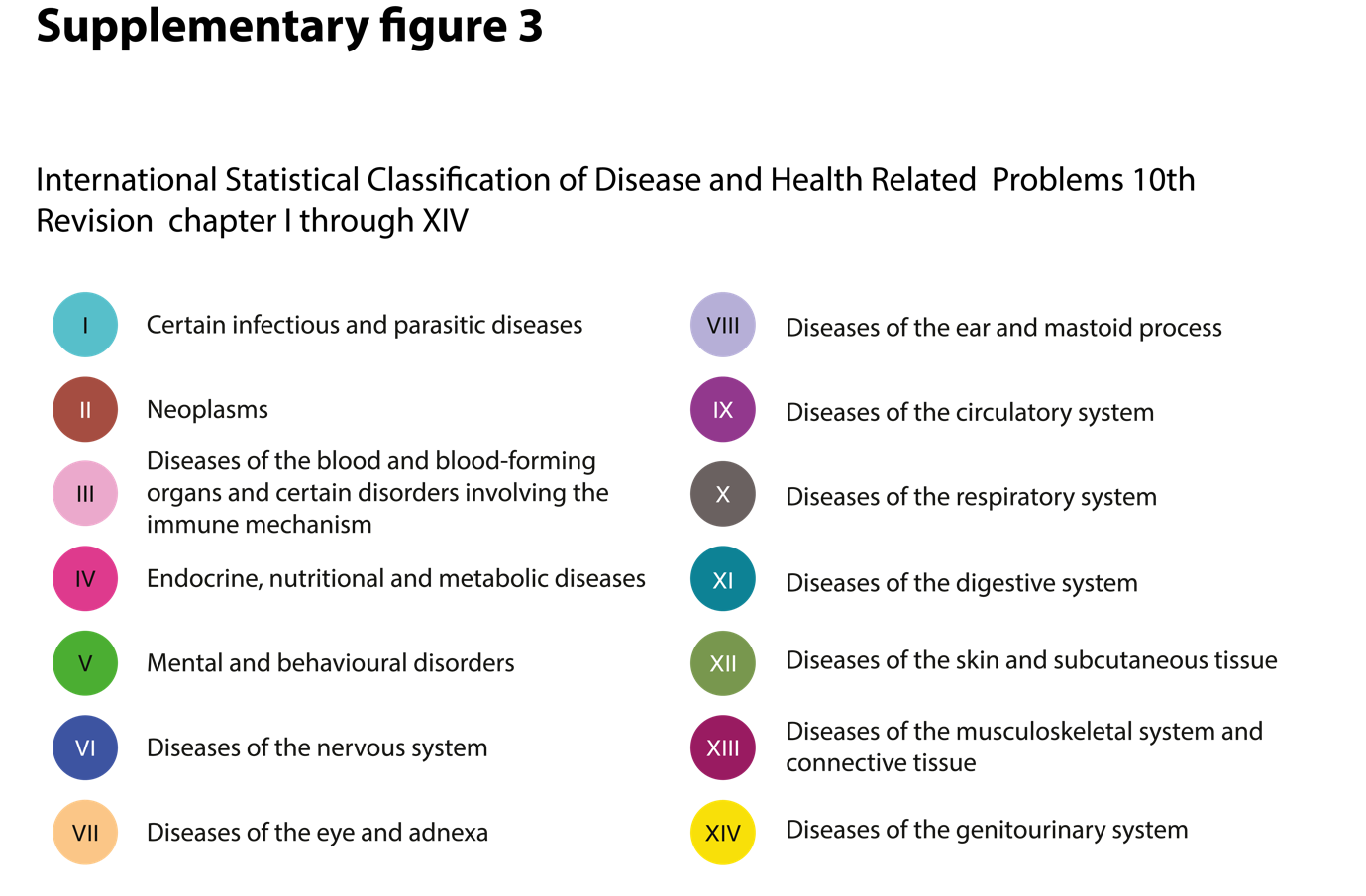

Supplement: Supplementary file 1 — Additional file 1: Fig. S1. Distribution of coefficients for covariates in the multiple linear regressions. Regression coefficients for the covariates type of patient (in- or out-patient), type of diagnosis (primary or non-primary diagnosis code), and sex (male or female). One observation per evaluated diagnosis and all diagnoses were evaluated in each plot. Fig. S2. Disease trajectory network pieced together from length two trajectories. Circles represent diagnosis codes (ICD-10 codes), and arrows represent a length two trajectory. IHD risk factors such as type 2-diabetes (E11) and hypertension (I10) appear in the left side of the graph. Angina pectoris (I20), acute myocardial infarction (I21), and chronic IHD (I25) have many incoming as well as outgoing edges consistent with the wide phenotypic spectrum they represent. ICD-10: International Statistical Classification of Diseases and Related Health Problems 10th Revision. IHD: Ischemic heart disease. For a full list of ICD-10 code definitions see Table S1. Fig. S3. Color key according to ICD-10 chapter. ICD-10: International Statistical Classification of Diseases and Related Health Problems 10th Revision chapter I through XIV. Table S1. ICD-10 codes and descriptions. [file 12933_2022_1527_MOESM1_ESM.docx]
